# Deletion of the RNA-editing enzyme ADAR1A: new strategy to potentiate responses to PD-1 immune checkpoint blockade

**DOI:** 10.1038/s41392-019-0039-8

**Published:** 2019-03-22

**Authors:** Manni Wang, Xiawei Wei

**Affiliations:** Laboratory of Aging Research and Cancer Drug Target, State Key Laboratory of Biotherapy and Cancer Center, National Clinical Research Center for Geriatrics, West China Hospital, Sichuan University, No. 17, Block 3, Southern Renmin Road, Chengdu,, 610041 Sichuan People’s Republic of China

**Keywords:** Cancer therapy, Lung cancer

In a recent study in *Nature*, Ishizuka et al.^1^ proposed a new strategy to restore sensitivity to immune checkpoint blockades (ICBs) through the loss of RNA-editing enzyme ADAR1 (adenine deaminase acting on RNA 1). This finding identifies ADAR1 as an attractive target to improve the treatment response in ICB-resistant patient.

Current knowledge on the interaction networks of immune checkpoint molecules, such as the intracellular PD-L1 “signalosome”, is far from conclusive.^2^ Despite the previous success of ICBs, more efforts are needed to overcome the therapeutic resistance to ICBs.

In a previous study, Manguso et al. used a CRISPR–Cas9 genome-editing approach in mice treated with immunotherapy to identify genes that are related to ICB resistance. A recent study published in *Nature* by Ishizuka et al. proposed a potential mechanism for overcoming ICB resistance through the loss of ADAR1. A-to-I editing refers to the process in which ADAR1 catalyzes the deamination of adenosine (A) to produce inosine (I), leading to the destabilization of double-stranded (ds) RNAs.^3^ The dsRNA has long been recognized as a trigger of innate immune responses including the release and action of interferon (IFN). IFN-stimulated genes are significantly upregulated in tumor cells following the deletion of ADAR1.^4^ On the basis of the association between IFN signaling and ADAR1, it is intriguing to speculate the regulatory roles of ADAR1 in innate immunity.

Ishizuka and his team^1^ first found that multiple types of tumors with ADAR 1 deletion were significantly sensitized to anti-PD-1 (programmed cell death protein 1) treatment. They found a marked increase in CD8+ T cells and the enhanced expression of IFN-associated genes in immune cells, suggesting that ADAR 1 deletion could promote the abundance of IFNs and at the same time, reshape the tumor immune microenvironment. The team then questioned whether the enhanced sensitivity of Adar1-null tumors to T cell killing was a result of the increased sensitivity to IFNs. Upon the stimulation of either IFNβ or IFNγ, Adar1-null tumors exhibited increased secretion of IFNβ and decreased cell viability. On the basis of the observation that only the deletion of both *Ifnar2* and *Ifngr1* genes was able to impair tumor responses to anti-PD-1 treatment, IFN sensing was crucial to restore the ICB sensitivity. They also found that the ADAR1 loss restored the sensitivity to PD-1 blockades through several common mechanisms for immunotherapy resistance. The loss of ADAR1 also led to a higher number of immune cells in B2m-null tumors, reshaping the tumor microenvironment without the T cell recognition of MHC I on tumors. The proposed model for ADAR1 deletion to increases the sensitivity to ICBs is presented in Fig. 1.

**Fig. 1:**
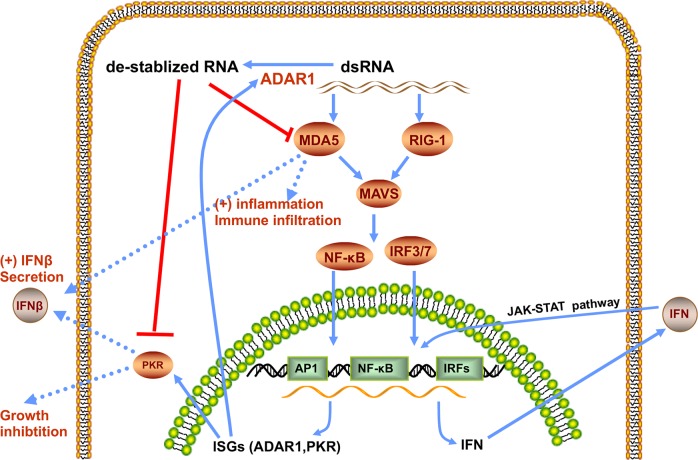
The proposed model for ADAR1 deletion to increases the sensitivity to ICBs

A previous study tested up to 2368 genes in tumor cells and found that the deficiency in IFN-γ signaling largely correlated with immunotherapy resistance. Furthermore, the loss of protein tyrosine phosphatase (PTPN2) in cancer cells promoted IFN-γ-mediated tumor antigen presentation and therefore improved the efficacy of immunotherapy. IFN can be produced by various cells in the tumor microenvironment and has been reported to augment innate immune reactions through the MDA5 and protein kinase R (PKR) pathways.^5^ However, its frequent therapeutic resistance limits its further application. One known mechanism for this resistance is deficient IFN signaling caused by the loss of ISG STAT1.^6^ ADAR1 deletion upregulates STAT1 and thus restores the sensitivity of tumor cells to IFN treatment. The concomitant use of ADAR1 blockade and IFN therapy therefore provides an additional treatment option for cancer patients.

Overall, these studies provide new insights into immune activations that do not necessarily require recognition between tumor cells and CD8+ T cells. The absence of tumor-specific T cell responses can be overcome by IFN-induced inflammation in tumors with ADAR1 loss. However, it has to addressed that currently no drug that specifically inhibits ADAR1 is available to decrease the hyper-editing events in tumor progression, and that the roles of other ADAR family members such as the ADAR2 and ADAR3 in ICB resistance still need further study. Another thing to be noted is that the simultaneous deletion of the two ADAR1 isoforms can lead to the embryonic lethality, characterized with the failure of hematopoiesis and organ development.^4^ Therefore, the ADAR1 deletion strategy still needs to overcome its potential toxicity to become a target in future cancer therapy. Despite all this, the recent finding identifies ADAR1 as an attractive target to restore the sensitivity to ICBs in cancer patients.

## References

[1] Ishizuka JJ (2019). Loss of ADAR1 in tumours overcomes resistance to immune checkpoint blockade. Nature.

[2] Escors D (2018). The intracellular signalosome of PD-L1 in cancer cells. Signal Transduct. Target Ther..

[3] Bass BL (2002). RNA editing by adenosine deaminases that act on RNA. Annu. Rev. Biochem..

[4] Hartner JC, Walkley CR, Lu J, Orkin SH (2009). ADAR1 is essential for the maintenance of hematopoiesis and suppression of interferon signaling. Nat. Immunol..

[5] Chung H (2018). Human ADAR1 prevents endogenous RNA from triggering translational shutdown. Cell.

[6] Bromberg JF, Horvath CM, Wen Z, Schreiber RD, Darnell JE (1996). Transcriptionally active Stat1 is required for the antiproliferative effects of both interferon alpha and interferon gamma. Proc. Natl Acad. Sci. USA.

